# The Future of Implementation Science for Public Health and Healthcare: Insights From the Swiss Implementation Science Network (IMPACT) Conference 2024

**DOI:** 10.3389/ijph.2026.1609478

**Published:** 2026-04-28

**Authors:** Sabina M. De Geest, Aita Signorell, Sarah Serhal, Kaspar Wyss, Marina Boccardi, Juliane Mielke, Suzanne Dhaini, Bastiaan Van Grootven, Sophie Gendolla, Christina Akre, Carole E. Aubert, Thekla Brunkert, Lauren Clack, Guy Haller, Cedric Mabire, Kate Molesworth, Aimad Ourahmoune, Jürg Utzinger, Marie Schneider

**Affiliations:** 1 Nursing Science, Department of Public Health, University of Basel, Basel, Switzerland; 2 Academic Center for Nursing and Midwifery, Department of Public Health and Primary Care, KU-Leuven, Leuven, Belgium; 3 Swiss Tropical and Public Health Institute, Allschwil, Switzerland; 4 University of Basel, Basel, Switzerland; 5 School of Pharmaceutical Sciences, University of Geneva, Geneva, Switzerland; 6 Institute of Pharmaceutical Sciences of Western Switzerland, University of Geneva, Geneva, Switzerland; 7 University of Applied Sciences and Arts of Southern Switzerland ‐ SUPSI, Manno, Switzerland; 8 University Children’s Hospital Zurich – Eleonore Foundation, Zürich, Switzerland; 9 Institute for Implementation Science in Health Care, University of Zurich, Zurich, Switzerland; 10 Unisanté, Center for Primary Care and Public Health, University of Lausanne, Lausanne, Switzerland; 11 Department of General Internal Medicine, Bern University Hospital, University of Bern, Bern, Switzerland; 12 Institute of Primary Health Care, University of Bern, Bern, Switzerland; 13 Faculty of Health Sciences and Medicine, University of Luzern, Luzern, Switzerland; 14 Department of Infectious Diseases and Hospital Epidemiology, University Hospital Zurich, Zurich, Switzerland; 15 Geneva University Hospitals‐Quality of Care Division, Faculty of Medicine, University of Geneva, Geneva, Switzerland; 16 Institute of Higher Education and Research in Healthcare, University of Lausanne, Lausanne University Hospital, Lausanne, Switzerland; 17 Independent, Formerly Swiss Tropical and Public Health Institute, Allschwil, Switzerland

**Keywords:** implementation science, interprofessional collaboration, public health, research infrastructure, research translation

## Introduction

Founded in 2019, the Swiss Implementation Science Network (IMPACT; https://impact-dph.unibas.ch) aims to strengthen the systematic uptake of evidence into healthcare and public health practice in Switzerland and beyond [1–3]. By fostering collaboration across research, policy, public health, clinical care, and communities, IMPACT seeks to improve population health, reduce research waste, and accelerate the translation of innovations into real-world settings. Implementation science (IS) is the scientific study of methods that promote the integration of evidence-based interventions into routine practice [4]. Core IS approaches include contextual analysis of multilevel barriers and facilitators (e.g., assessment of readiness for change [5, 6], continuous stakeholder engagement and co-creation, theory- and contextual-informed implementation strategies, theory- and contextual-informed intervention development/adaptation, and evaluation of both effectiveness and implementation outcomes [2].

At the third IMPACT conference in Geneva in November 2024, experts and 126 participants discussed how IS can enhance the societal impact of research and support timely, equitable uptake of evidence across healthcare and public health. This paper summarizes key insights from the roundtable on positioning IS within research infrastructures, together with findings from an interactive poll on expectations of the IMPACT network. We highlight the potential of IS to improve healthcare and outline priorities to strengthen its capacity and infrastructure in Switzerland.

## The Critical Role of Implementation Science for Healthcare and Public Health

A substantial proportion of health research fails to translate into practice, delaying benefits for patients and populations [7–9]. Implementation science holds promise to bridge the gap between research findings and real-world application by offering methods for timely knowledge translation. It is widely acknowledged that implementation science should be integrated early - ideally during the development of innovations such as medications, technologies, or services across all domains of healthcare, including public health. As one conference panelist emphasized, the question is not whether implementation science is important, but rather, “*How can we survive without implementation science?*”.

Implementation science is vital for public health, where change occurs at the level of populations, systems, and policies rather than individual patients. Its methods can speed the adoption of preventive interventions, screening programs, vaccination strategies, and health promotion policies. Recent articles in the *International Journal of Public Health* highlight this scope, from organized colorectal cancer screening in Switzerland [10] and the CHESS mixed-methods HPV screening intervention for women living with HIV in Nigeria [11] to translational work on healthy aging [12]. Together, these examples show that effective public health implementation requires broader methodologies that explicitly address real-world translation. Strengthening implementation science infrastructure will therefore advance not only clinical care but also prevention, population health, and global health efforts.

## Challenges to Integrating Implementation Science Into Healthcare and Public Health

**FIGURE 1:**
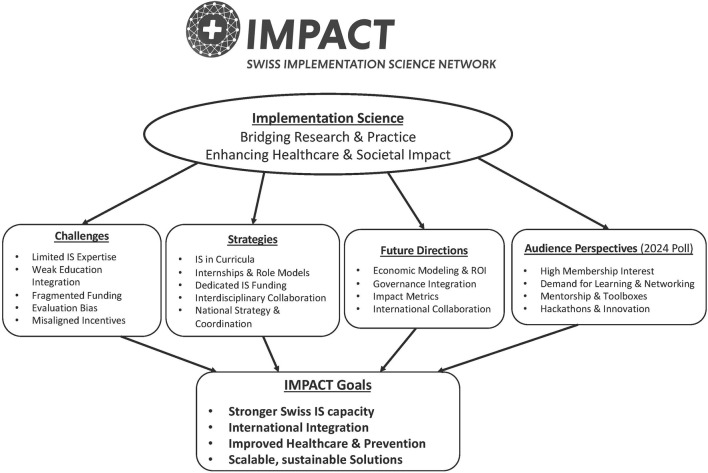
Summary of key findings and insights of panel and poll of the 2024 Swiss Implementation Science Network (IMPACT) conference in Geneva, Switzerland *IS: implementation science, ROI return on investment*.

Despite its recognized importance, implementation science faces several barriers to integration within existing research infrastructures, healthcare systems, and society [13]. Panelists identified a number of them with the main ones being that (Figure 1): (i) many scientists lack experience in translating innovations into real-world practice and managing the financial responsibilities aligned with societal priorities; (ii) implementation science principles are not yet fully embedded in standard healthcare education, nor research methods training, leaving professionals without the skills needed to drive system transformation; (iii) funding for large-scale implementation science initiatives remains fragmented and often insufficient, especially compared to fundamental biomedical research; (iv) evaluation committees may also lack the expertise required to fairly assess implementation science projects, leading to potential bias against implementation science approaches; and (v) academic incentives based on the number of grants and publications over real-world impact of the results of a research project can further hinder the advancement of implementation science.

## Strategies for Advancing Implementation Science

Roundtable panelists from diverse health sciences backgrounds, as well as from a foundation, proposed several strategies to advance implementation science and address the identified barriers. These strategies include *(i) implementation science education and training; (ii) funding and rational resource allocation; (iii) inter- and transdisciplinary collaboration; and (iv) strategic development* (Figure 1).

### Education and Training

Panelists recommended integrating implementation science principles into healthcare curricula at all levels (BSc, MSc, PhD, and professional postgraduate education) through dedicated courses and practical examples. For example, the Swiss School of Public Health (SSPH^+^) has already integrated IS in their summer course program (https://www.ssph-lugano-summerschool.ch/). Panelists also proposed developing internships and placements specifically designed to provide researchers with hands-on experience in translating evidence into practice. Role modelling by experienced clinicians and public health experts, along with “train-the-trainer” programs that showcase implementation science success stories and case studies, were identified as effective approaches to strengthening training in implementation science. Education should emphasize both data collection to support informed decision-making and the development of skills for effective stakeholder engagement, including collaboration with policymakers and front-line implementers.

### Funding and Resource Allocation

To strengthen funding for implementation science, panelists proposed developing a national strategic roadmap for large, targeted implementation science grants, as well as integrating such funding into existing programs. They also encouraged seeking alternative funding sources beyond traditional research grants and demonstrating the value and competitiveness of implementation science to attract broader support.

### Inter- and Transdisciplinary Collaboration

Building ecosystems that connect research, clinical practice, public health, patients, decision-makers, industry, and education was considered essential. For example, greater efforts are needed to ensure that implementation strategies tested in implementation science projects are adequately supported and adopted by practitioners and health systems, enabling them to leverage proven approaches to foster changes in practice and, ultimately, reduce research waste. Encouraging dialogue with IS skeptics and critics was also highlighted as a way to broaden understanding and support for the field.

### Strategic Development

Panelists recommended establishing a clear, long-term strategy for the Swiss IMPACT network, with measurable goals and key performance indicators that reflect stakeholder input. Suggested actions included developing a national implementation science roadmap, appointing a network coordinator, and making implementation science a required component of research proposals. Panelists also emphasized the importance of developing case studies to demonstrate the societal value of implementation science and focusing on sustainable, scalable solutions to real-world problems. Expanding implementation science beyond clinical care to public health and prevention, was strongly encouraged, as illustrated by the Swiss colorectal cancer screening in Switzerland [10].

## Future Developments of Implementation Science

Based on the roundtable discussion with experts, several priorities emerged for the future of implementation science (Figure 1).

### Economic Evaluation and Policy Engagement

Developing robust health economic models, including cost–benefit analyses, to inform policymakers and decision-makers is essential. Such approaches can support the development of comprehensive economic frameworks that capture system-level impacts, thereby enabling a deeper understanding of the nuanced value of implementation science.

### Leadership, Governance, and Academic Incentives

Implementation science principles should be integrated at higher levels of healthcare organizations and public health by educating leaders about the complexity of implementation processes and the waste of resources that occurs when implementation fails. Panelists also recommended building new ecosystems that connect academia, clinical practice, public health, and education, similar to the Medical Research Council (MRC) Centres of Research Excellence in the United Kingdom.

### Standardization, Evaluation, and International Collaboration

To further establish the value of implementation science, standardized metrics for measuring real-world and societal impact should be developed, alongside rigorous evaluation of large-scale implementation science initiatives. Success stories, as well as lessons learned from failures, should be widely shared. The field would also benefit from increased international collaboration, including the sharing of best practices, the development of global standards and guidelines, and the establishment of international networks of implementation science researchers, public health experts and clinical practitioners.

## Audience Perspectives on How to Further Develop IMPACT

Results from an interactive poll (i.e., Mentimeter) conducted at the 2024 IMPACT Conference in Geneva highlighted growing interest in the network and its mission (Figure 1). Of the 77 respondents (representing 61% of the 126 participants), more than half expressed interest in becoming members, indicating a strong foundation for future growth.

Participants showed the greatest enthusiasm for interactive and educational activities, such as conferences, webinars, and courses on implementation science, reflecting a clear demand for knowledge-sharing and skill-building opportunities. There was moderate interest in more hands-on involvement, including participation in methodology development projects and exploration of funding opportunities. The Swiss Implementation Science repository and YouTube channel received lower ratings; however, this may be explained by the relatively recent launch of these tools.

Our findings echo feedback from the first IMPACT conference in 2021 [3], which emphasized the need for mentorship, practical training, and accessible resources. As in 2021, participants in 2024 stressed the importance of networking, collaboration, and internationalization. Specific suggestions included establishing mentorship programs, offering practical courses with step-by-step manuals, developing implementation science toolboxes, organizing topic-specific networking events, and increasing support for international networking [13].

Finally, attendees emphasized the importance of practical application, stakeholder engagement, and innovative approaches—such as hackathons and co-developed vision statements—as essential for advancing IMPACT’s goals.

### Conclusion

Implementation science is a critical enabler of effective public health and preventive action. By addressing contextual determinants, strengthening system readiness, investing in stakeholder involvement and aligning research with societal needs, it can accelerate the translation of evidence into sustainable population health gains across the life course.

The IMPACT network provides a strategic infrastructure to build national capacity, foster collaboration, and demonstrate the real-world value of implementation science. Continued investment in education, governance, and cross-sector partnerships will be essential to reduce research waste and deliver scalable, equitable, and preventive health improvements.

## References

[1] De Geest S Akre C Aubert C Brauchli P Brunkert T Dhaini S Accelerating Innovation: Implementation Science as a Cornerstone of High-Performance Swiss Research Infrastructures. Swiss Med Wkly (2025) 155:4501. 10.57187/s.4501 41100820

[2] De Geest S Zúñiga F Brunkert T Deschodt M Zullig LL Wyss K Powering Swiss Health Care for the Future: Implementation Science to Bridge The Valley of Death. Swiss Med Wkly (2020) 150:w20323. 10.4414/smw.2020.20323 33022070

[3] Dhaini SR Mielke J Brunkert T Wyss K Utzinger J De Geest S . Swiss Implementation Science Network (IMPACT): A Crucial Building Block to Strengthen the Swiss Research Pipeline for Real-World Translation. Int J Public Health (2021) 66:1604081. 10.3389/ijph.2021.1604081 34335151 PMC8284855

[4] Eccles MP Mittman BS . Welcome to Implementation Science. Implement Sci (2006) 1(1):1. 10.1186/1748-5908-1-1

[5] Weiner BJ . A Theory of Organizational Readiness for Change. Implement Sci (2009) 4:67. 10.1186/1748-5908-4-67 19840381 PMC2770024

[6] Andersen JB Gulis G . Community Maturity to Implement Health in all Policies. Int J Public Health (2017) 62(5):605–12. 10.1007/s00038-017-0951-z 28224213

[7] Jackson GL Cutrona SL Kilbourne A White BS Everett C Damschroder LJ . Implementation Science: Helping Healthcare Systems Improve. JAAPA (2020) 33(1):51–3. 10.1097/01.JAA.0000615508.92677.66 31880652

[8] Rubin R . It Takes an Average of 17 Years for Evidence to Change Practice - the Burgeoning Field of Implementation Science Seeks to Speed Things up. JAMA (2023) 329(16):1333–6. 10.1001/jama.2023.4387 37018006

[9] Wensing M Wilson P . Making Implementation Science More Efficient: Capitalizing on Opportunities Beyond the Field. Implement Sci (2023) 18(1):40. 10.1186/s13012-023-01298-9 37697372 PMC10496297

[10] Albers B Auer R Selby K Clack L . Organized Colorectal Cancer Screening Programs in Switzerland - Quo Vadis? Int J Public Health (2025) 70:1608183. 10.3389/ijph.2025.1608183 40302766 PMC12038373

[11] Ogunsola O Gaydos LM Ajayi O Dieci M Kaonga N Awolude O The CHESS Protocol: A Mixed-Methods Evaluation of an HPV Screening Intervention for Women Living with HIV in Nigeria. Int J Public Health (2025) 70:1608716. 10.3389/ijph.2025.1608716 40895387 PMC12391886

[12] Solano AHJ . Translational Research and Implementation of Solutions for Aging: A Call to Action. Int J Public Health (2025) 70:1607768. 10.3389/ijph.2025.1607768 40365335 PMC12069069

[13] Schultes M-T Finsterwald M Brunkert T Kien C Pfadenhauer L Albers B . Barriers and Facilitators for Conducting Implementation Science in German-Speaking Countries: Findings from the Promote Impsci Interview Study. Glob Implement Res Appl (2022) 2(2):120–31. 10.1007/s43477-022-00046-3 35637900 PMC9134978

